# Noninvasive Mechanochemical Imaging in Unconstrained *Caenorhabditis elegans*

**DOI:** 10.3390/ma11061034

**Published:** 2018-06-19

**Authors:** Takuma Sugi, Ryuji Igarashi, Masaki Nishimura

**Affiliations:** 1 Molecular Neuroscience Research Center, Shiga University of Medical Science, Otsu, Shiga 520-2192, Japan; mnishimu@belle.shiga-med.ac.jp; 2 PRESTO, Japan Science and Technology Agency, 4-1-8 Honcho, Kawaguchi, Saitama 332-0012, Japan; 3 QST Future Laboratory, National Institute for Quantum and Radiological Science and Technology, Anagawa 4-9-1, Inage-ku, Chiba 263-8555, Japan; 4 Department of Molecular Engineering, Graduate School of Engineering, Kyoto University, Nishikyo-ku, Kyoto 615-8510, Japan

**Keywords:** *C. elegans*, behavior, piezoelectric actuator, mechanochemistry, calcium imaging

## Abstract

Physical forces are transduced into chemical reactions, thereby ultimately making a large impact on the whole-animal level phenotypes such as homeostasis, development and behavior. To understand mechano-chemical transduction, mechanical input should be quantitatively delivered with controllable vibration properties–frequency, amplitude and duration, and its chemical output should be noninvasively quantified in an unconstrained animal. However, such an experimental system has not been established so far. Here, we develop a noninvasive and unconstrained mechanochemical imaging microscopy. This microscopy enables us to evoke nano-scale nonlocalized vibrations with controllable vibration properties using a piezoelectric acoustic transducer system and quantify calcium response of a freely moving *C. elegans* at a single cell resolution. Using this microscopy, we clearly detected the calcium response of a single interneuron during *C. elegans* escape response to nano-scale vibration. Thus, this microscopy will facilitate understanding of in vivo mechanochemical physiology in the future.

## 1. Introduction

Animals receive time-varying mechanical inputs such as touch, vibration and gravity from their surrounding environment [1,2,3]. To survive these physical environments and ensure their reproductions, they process input information through computation in their neural circuit, thereby changing behavior. In the circuit, the mechanical information is transduced into chemical reactions such as calcium signaling and ATP hydrolysis at a single neuron level. Understanding this mechanochemical transduction, especially in terms of physiology of how much force frequency, amplitude and duration could evoke chemical output, requires quantitatively and precisely controlling vibration properties of mechanical input. In addition to the delivery of input stimuli, the quantification of a chemical output in an intact neural circuit was required. However, it has been often hampered by the invasive introduction of a chemical probe to measure intracellular dynamics and the constraint of animal body movement, because the introduction of a probe and constraint inevitably include mechanical stimulus per se. Thus, there have been hardly any experimental systems to noninvasively and unconstrainedly quantify the input-output relationship at a single neuron level.

Mechanosensory behavior in *C. elegans* is a simple behavioral paradigm, in which worms usually exhibit a withdrawal escape response to mechanosensory stimulus such as touch stimulus and nonlocalized vibration by tapping a cultivated Petri plate [4]. Furthermore, after spaced training for repeated stimulation, worms habituate to the stimulus and there are significant decreases in reversal distances over a long period (>24 h) [5,6]. In this paradigm, therefore, memory is easily quantified by comparing the reversal response between trained and untrained worms. Importantly, the neural circuit that underlies this memory-based behavior has been identified and primarily composed of five sensory neurons, four pairs of interneurons, and few motor neurons [4,7]. In recent studies, noninvasive recoding of neural activity has been achieved by calcium imaging of a freely moving worm that expresses the genetically encoded calcium indicator in a cell-specific manner [8,9,10,11]. This noninvasive imaging enables interrogating physiological aspects of each neuron within the neural circuit. However, in the context of mechanosensory physiology, it has been difficult to precisely and quantitatively control the vibration properties of mechanical stimulus presented to freely behaving worms.

We recently developed a new nonlocalized vibration device using a piezoelectric acoustic transducer [12]. Using this device, we demonstrated how to flexibly change the vibration properties at a nanoscale displacement. This development motivated us to integrate this piezoelectric system with a noninvasive calcium imaging system. In this study, we show mechanochemical calcium imaging in *C. elegans*. We used a piezoelectric acoustic transducer to evoke nonlocalized vibration with flexibly tuned vibration parameters. The piezoelectric system was integrated into a system for calcium imaging of freely moving worms. As proof of principle, we clearly quantified calcium response of worms that express GCaMP in the AVA interneurons under the control of vibration frequency. This analysis indicated that calcium influx in the AVA neuron is significantly higher at 630 Hz in comparison with those at other vibration frequencies. Thus, our integrated device will facilitate understanding of the physiological relationship between input nonlocalized mechanical stimulus and causal output calcium response in the future.

## 2. Materials and Methods

**Molecular Biology.** PCR was conducted to amplify a TagRFP cDNA and NLS-GCaMP5G-NLS from pTAK73 vector and pDEST-NLS-GCaMP5G-NLS, respectively. These amplicons flanked by Acc65I and AgeI restriction enzyme sites were each subcloned into the HincII-digested vector pBluescript KS (-) (Addgene). The DNA fragments encoding TagRFP and NLS-GCaMP5G-NLS were digested by Acc65I and AgeI, and then ligated into the pTAK50 vector that contains the 4.5 kb PCR-amplified *flp-18* promoter to generate pTAK83 plasmid and pTAK144 plasmid, respectively.

**Strain preparation.** Germline transformation was performed as described [13]. To generate ST10 strain, the obtained pTAK83 (20 ng/μL) was injected into the *lite-1(ce314)* strain, which is commonly used as a light insensitive mutant for calcium imaging experiments in *C. elegans* [14]. pTAK144 plasmid (20 ng/μL) and the injection marker *ges-1p::NLS-EGFP* (pKDK66; 50 ng/μL) are co-injected into wild-type worms to generate ST11 strain. The ST11 was outcrossed three times with ST10 strain to obtain ST12 strain that expresses TagRFP and NLS-GCaMP5G-NLS in the AVA neuron. All strains were maintained and handled using standard methods [15].

**Piezoelectric device.** To control the properties of nonlocalized vibration precisely, a piezoelectric system was constructed using a piezoelectric acoustic transducer (THRIVE, pzBAZZ μSpeaker B35) as an actuator and the amplifier module (THRIVE, 0530AMPZ) connected to a computer earphone jack via an earphone splitter. The diameter of the transducer was 42 mm. A free download software, WaveGene Ver. 1.50, was used for the control of vibration properties.

**Calcium imaging.** The system consists of an Olympus MVX10 dissection scope equipped with a 2× objective lens (MVPLAPO2XC; WD 20 mm and NA 0.5), high-speed Zyla CMOS camera (Andor, Belfast, UK), W-VIEW GEMINI Image splitting optics (HAMAMATSU Photonics, Hamamatsu, Japan), x-y motorized stage (Thorlabs, Newton, NJ, USA) and computer (Dell, Round Rock, TX, USA). GCaMP and TagRFP were excited by a mercury lamp filtered at 451–485 nm and 541–565 nm, respectively, using a Semrock dual-bandpass filter. The fluorescence image was split by a W-VIEW GEMINI Image splitting optics, and the two images (green channel, 500–525 nm; red channel, 584–676 nm) were projected onto two halves of a CMOS camera. The real-time processing of the fluorescent images was achieved by the custom-made machine vision software written in LabVIEW (National Instruments, Austin, TX, USA). This software also controls the x-y motorized stage and follows a fluorescent object of a worm in dark field by its brightness. In particular, a feedback loop system is used to track the fluorescent spot by instructing the stage to move the spot to the center of the camera field. Images were acquired with 0.041 ms exposure time with 2 × 2 binning. The focus was adjusted manually. The machine vision software ran on a Dell precision T7600 computer running Windows 7 with Intel Xeon CPU ES-2630 and 8 GB of RAM.

Worms were grown to adult stage on a 60 mm Petri plate (Thermo Fisher Scientific, Waltham, MA, USA, #150288) containing 10 mL of nematode growth medium (NGM) with agar, on which *Escherichia coli* OP50 was seeded. Just before mechanochemical imaging, a single worm was transferred to a new fresh NGM surface covered with a thin layer of *Escherichia coli* OP50. The NGM plate was gently placed on the actuator at 1.5 min before tracking. Then, calcium imaging was performed for the freely moving worm without any physical restraint. The fluorescent spot of each worm was tracked under the TagRFP channel.

Image analysis was performed using ImageJ and Mathematica 9.0 (Wolfram, Champaign, IL, USA). Briefly, at first, the coordinate in which the intensity of TagRFP fluorescence was maximum after background subtraction was extracted for each image. This TagRFP signal serves to compensate for focus changes caused by the animal’s motion. Then, the maximum fluorescent intensity of GCaMP within the extracted coordinate ± 10 pixels was calculated as GCaMP signal intensity for each image. For ratiometry, (I_GCaMP_ − I_GCaMP__BG)/(I_TagRFP_ − I_TagRFP__BG) was calculated, in which I_GCaMP_ and I_TagRFP_ are the GCaMP and TagRFP signals from the AVA neuron, respectively and I_GCaMP__BG and I_TagRFP__BG are the background signals, respectively. This calculation process was applied for all images to quantify the ratio change in a reversal event.

## 3. Results

### 3.1. Device Design

**Figure 1:**
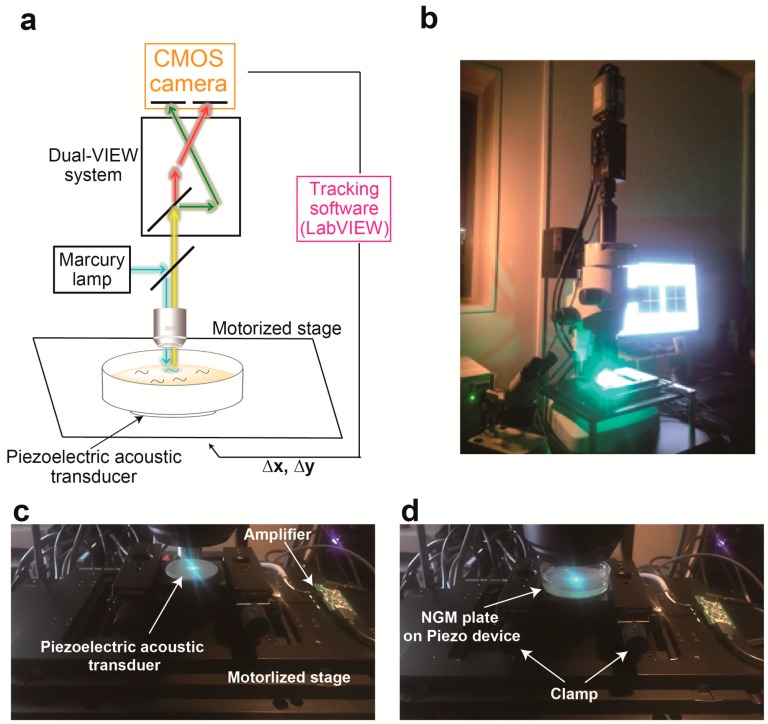
A system integrating piezoelectric device into calcium imaging system (**a**) Schematic diagram of the experimental set-up for the calcium imaging system combined with piezoelectric device. (**b**) The photograph of a new system. (**c**,**d**) The close-up photo of a piezoelectric device attached on the motorized stage (**c**) and an NGM plate on the actuator (**d**).

**Figure 2:**
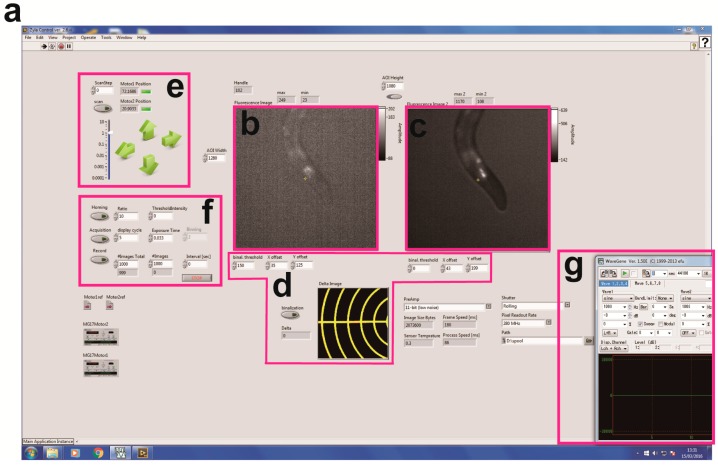
Screenshot for the machine vision software and each control panel. (**a**) Overall photo of the software written on LabVIEW. (**b**,**c**) GCaMP (**b**) and TagRFP (**c**) images. (**d**) A panel for calibration of the coordinates between GCaMP and TagRFP images. (**e**) A panel for controlling the X-Y position of motorized stage. The coordinate is recorded as a csv file. The stage can be also driven by screw actuators under joystick control (Thorlabs, Newton, NJ, USA). (**f**) A panel for image acquisition and tracking. Exposure time, binning and image acquisition rate can be changed in this panel. (**g**) A photo for WaveGene software to control vibration properties.

We initially designed a calcium imaging device using a motorized x-y translation stage (Figure 1). The NGM plate on which worms cell-specifically expressed NLS-GCaMP5G-NLS as a calcium indicator and TagRFP as a calcium-insensitive fluorescent reference was placed on the motorized stage. To enable ratiometric imaging, we used a W-VIEW GEMINI Image splitting optics to simultaneously image GCaMP and TagRFP signals side-by-side, and recorded image sequences using a high-speed CMOS camera (Figure 1a,b). The software was written in LabVIEW (Figure 2) so that each frame of the acquired images was subjected to real-time processing to detect targeted fluorescent spot (Figure 2b), track them (Figure 2f), record stage positions (Figure 2e), and re-center the tracked cells. Furthermore, we made it possible to set a schedule to record the image into the computer (Figure 2f). The maximum recording rate is 24.4 frames per second. Because the tracking rate is limited by the image processing, the maximum rate of the tracking is the same as that of the recording.

We then tried to construct a nonlocalized vibration device on the calcium imaging system. Initially, we designed the tap stimulation system using the cylinder and actuator, because this method was successfully applied to the quantification of tap habituation behavior in our and other groups [5,16]. However, we also confronted the problem that tapping a Petri plate on the motorized x-y stage caused out-of-view field of the fluorescent spot, leading to failure in tracking a freely behaving worm.

To avoid this out-of-view field problem upon stimulation, we adopted a recently established method to evoke nonlocalized vibration on an NGM plate [12] (Figure 1c,d). In this method, a circular-shaped piezoelectric acoustic transducer is used as an actuator that vibrates across the *z*-axis. Amplitude of vibration can be set by the volume control of a computer, and this sound volume level was changed in the range of 0 to 100%. On the other hand, the free download software was used for setting the frequency and duration of the vibration (Figure 2g). The frequency of vibration can be changed in the range of 0 to 2 kHz. The minimum duration of stimulation is 0.1 s. Moreover, this device allows for generating various waveforms such as sine wave, square wave, pulse wave and white noise. We previously demonstrated that this method allows for changing vibration properties of mechanical stimulus at a nanoscale displacement level [12]. The piezoelectric device was attached on the surface of the motorized x-y translation stage (Figure 1c), and an NGM plate on which worms freely moved was clamped using carriers for optical rail (Sigma Koki, Tokyo, Japan, CAA-25LSEE) and an optical baseplate (Sigma Koki, Tokyo, Japan, A50-4) on the device (Figure 1d).

### 3.2. Proof of Princple Experiment

**Figure 3:**
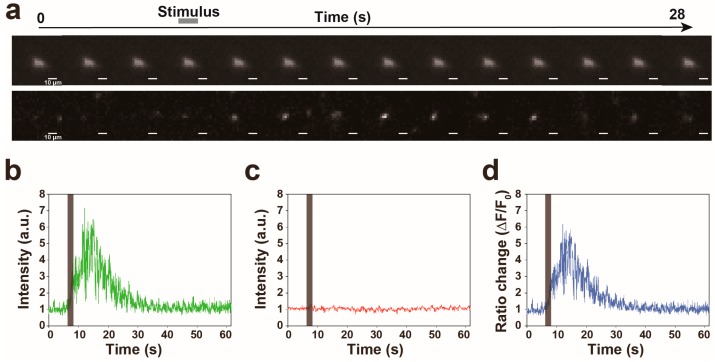

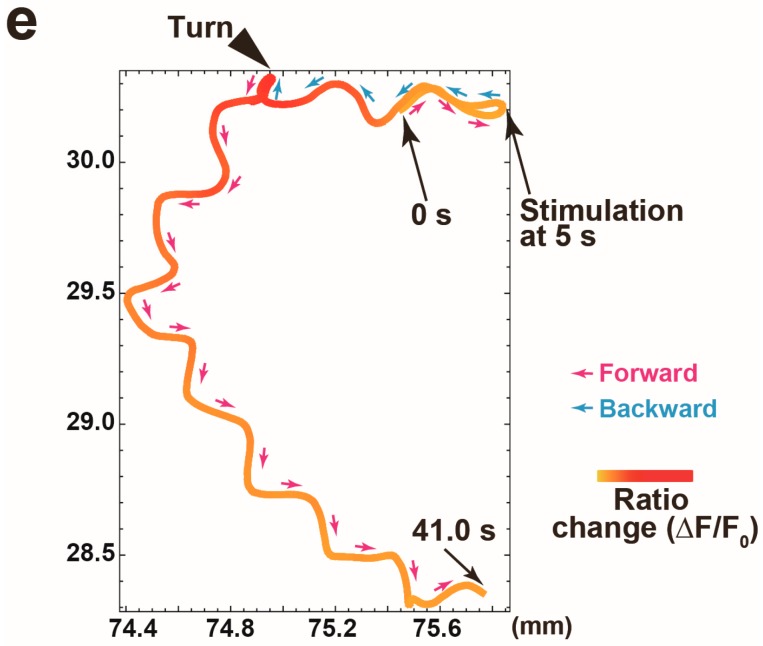
Proof of principle experiment for quantification of calcium signal in the AVA neuron while a worm responds to 1 kHz of nonlocalized vibration. (**a**) The representative filmstrip showing a GCaMP signal of the AVA neuron in a worm responding to nonlocalized vibration. Frames displayed here are separated by 2 s, but actual time resolution is higher. (**b**–**d**) The representative time traces of the intensities of GCaMP (**b**) and TagRFP (**c**) fluorescences and the ratio change (**d**) in a worm responding to nonlocalized vibration. (**e**) A trajectory of a worm with a calcium signal intensity of the AVA neuron indicated in (**d**). The arrowhead indicates the end of backward movement. The position where nonlocalized vibration was evoked is indicated as ‘Stimulation’.

To validate this microscopy, we cultivated worms that express GCaMP in the AVA interneurons using the *flp-18* promoter in an NGM plate, because the calcium level of the AVA neuron has been proven to increase during reversals evoked by nonlocalized vibration [5,7]. This NGM plate was placed on the actuator. We next evoked nonlocalized vibration with 1 kHz of frequency, 100% of computer sound volume level and 1 s of duration and tried to simultaneously record the fluorescent signals of GCaMP and TagRFP in a worm during reversal response. As shown in Figure 3a, we succeeded in tracking the fluorescence of TagRFP in the freely moving worm. Furthermore, quantifications of fluorescent signals of GCaMP and TagRFP have revealed that the fluorescent signal of GCaMP transiently increased during reversal responses (Figure 3b) but that of TagRFP did not change (Figure 3c), suggesting that the change of calcium signal was clearly detected in worms responding to nonlocalized vibration by our new imaging device (Figure 3d,e). The representative imaging data was indicated in Video S1, in which the calcium transient of the AVA neuron was clearly detected in a freely moving worm in response to 630 Hz of nonlocalized vibration.

### 3.3. Calcium Responses of the AVA Neuron to Nonlocalized Vibration with Different Frequencies

**Figure 4:**
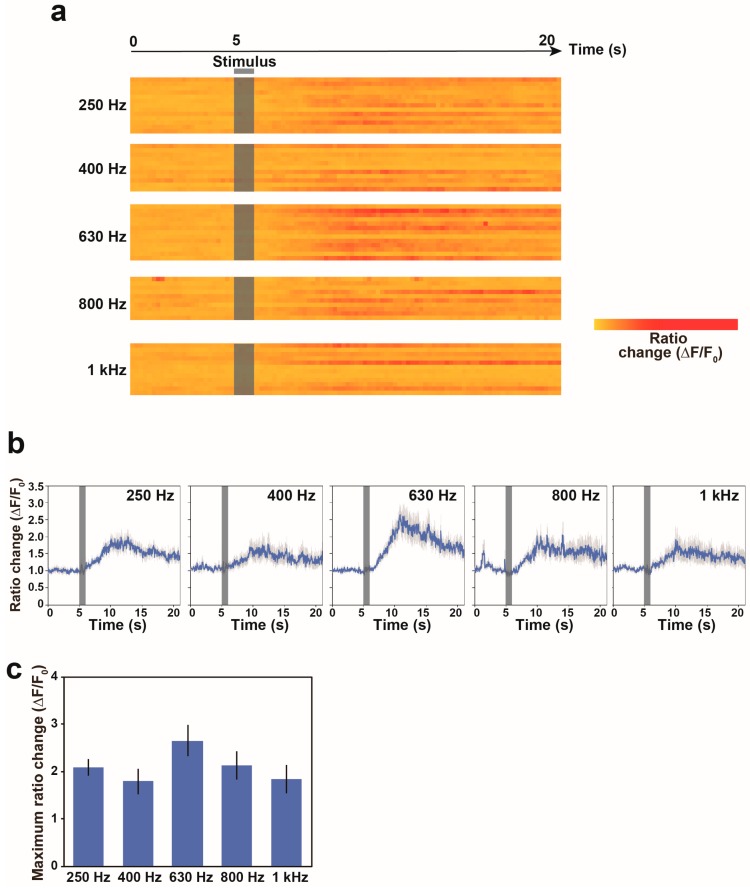
Calcium responses of freely behaving worms to nonlocalized vibrations with different vibration frequencies. (**a**) Heat maps showing calcium signals in the AVA neuron of freely behaving worms in response to stimulation. Each row in the heatmap shows the ratio change in a single worm. The ratio change in each bin was normalized by the maximum intensity observed in the worm responding to 630 Hz vibration. Nonlocalized vibration was evoked for 1 s and indicated as a black bar. Each line indicates a calcium response trace of the AVA neuron in one worm. (**b**) The average calcium responses of the AVA neuron in freely moving worms in response to nonlocalized vibrations. Error bars indicate standard error of the mean (s.e.m.). (**c**) The maximum calcium response of the AVA neuron in response to different nonlocalized vibrations. N = 13 worms in 250 Hz, 11 worms in 400 Hz, 13 worms in 630 Hz, 10 worms in 800 Hz and 12 worms in 1 kHz. Error bars indicate standard error of the mean (s.e.m.).

We measured the calcium responses of the AVA neurons under the control of vibration frequencies. The nonlocalized vibrations were evoked at 250 Hz, 400 Hz, 630 Hz, 800 Hz and 1 kHz of vibration frequencies for 1 s. The applications of these different vibration frequencies to the system were performed in random order. As shown in Figure 4a,b, our analysis suggests that the AVA neuron largely or slightly responds to nonlocalized vibration at all examined frequencies. The average calcium responses of the AVA neuron were 7.3, 6.3, 12.2, 7.2 and 4.0 at 250 Hz, 400 Hz, 630 Hz, 800 Hz and 1 kHz frequencies of nonlocalized vibrations, respectively (Figure 4c). These results indicated that calcium response in the AVA neuron is significantly higher at 630 Hz in comparison with those at other vibration frequencies, which is suggested to be the maximum calcium response in the AVA neuron.

## 4. Discussion

Compared with local touch stimulation [17,18], the physiological response induced by nonlocalized vibration has been less investigated in not only nematodes but also in mammals. We have recently focused on nonlocalized vibration and established a new system that allows for controlling vibration properties at a nanoscale displacement level using Piezoelectric acoustic transducer [12]. To facilitate understanding of physiology for vibration that inevitably affects the entire body and metabolism, we here established the noninvasive and unconstrained mechanochemical imaging microscopy to analyze the relationship between nonlocalized mechanical input stimulus and output calcium response in *C. elegans*. Previous electrophysiological and behavioral analysis indicated that the sensitivity of the touch receptor neuron ALM to touch stimulus reaches maximum value at 725 Hz, around which the probability of the reversal response to ultrasound stimulus is also maximum [17,19]. The ALM neuron innervates with the downstream interneuron AVD and thereby indirectly regulates the AVA neuron. In addition, worms whose ALM neuron was ablated by laser exhibit almost no reversal response to tap stimulus but accelerate forward movement, suggesting no direct influence of nonlocalized mechanical stimulus on the AVA neuron [20]. Therefore, consistent with these previous studies, our result suggests that the maximum value of calcium response observed in the downstream neuron AVA is similar to that of sensitivity in the ALM neuron. Our piezoelectric acoustic transducer provides a unique way to understand physiological functions of each neuron in the context of mechanosensation in the future.

## Supplementary Materials

The following are available online at http://www.mdpi.com/1996-1944/11/6/1034/s1. The source code of the software written in LabVIEW (Figure 2) can be downloaded. Video S1: The calcium transient of the AVA neuron detected in a freely moving worm in response to 630 Hz of nonlocalized vibration. Stimulation was evoked at 5.0 s. GFP was also expressed in intestine of the worm as the co-injection marker of GCaMP, and the fluorescence of GFP does not affect the signal intensity of GCaMP. The scale bar is also indicated in the first image. The moving directions of the worm are indicated at top left after the first image.
