# Supplementary figures and images for: Noninvasive Mechanochemical Imaging in Unconstrained Caenorhabditis elegans
Source: Materials (Basel). 2018 Jun 19;11(6):1034. doi: 10.3390/ma11061034 (PMC6025516; doi:10.3390/ma11061034)

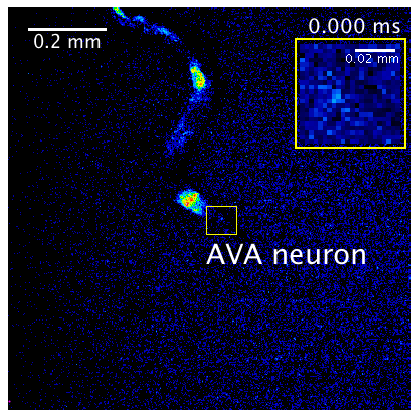

Supplement: Supplementary file 1 [file materials-11-01034-s001.zip › materials-315683/materials-315683-Video S1.gif]
